# LncRNA *CRNDE* Drives the Progression of Hepatocellular Carcinoma by inducing the Immunosuppressive Niche

**DOI:** 10.7150/ijbs.85471

**Published:** 2024-01-01

**Authors:** Xianying Li, Shuhang Liang, Mingming Fei, Kun Ma, Linmao Sun, Yao Liu, Lianxin Liu, Jiabei Wang

**Affiliations:** 1 Department of Hepatobiliary Surgery, The First Affiliated Hospital of USTC, Division of Life Sciences and Medicine, University of Science and Technology of China, Hefei, Anhui, China.; 2 Department of Gastrointestinal Surgery, The First Affiliated Hospital of USTC, Division of Life Sciences and Medicine, University of Science and Technology of China, Hefei, Anhui, China.; 3 Anhui Province Key Laboratory of Hepatopancreatobiliary Surgery, Hefei, Anhui, China.; 4 Anhui Provincial Clinical Research Center for Hepatobiliary Diseases, Hefei, Anhui, China.

**Keywords:** Hepatocellular carcinoma, Colorectal tumor differential expression, Tumor microenvironment, Myeloid-derived suppressor cell, C-X-C motif chemokine ligand 3

## Abstract

As a crucial protumorigenic long noncoding RNA, colorectal tumor differential expression (CRNDE) has been confirmed to facilitate the progression of various cancers. However, its role in the tumor microenvironment (TME) of hepatocellular carcinoma (HCC) is still unclear. Here we determined that CRNDE was upregulated in HCC samples and that CRNDE-positive cells were predominantly enriched in malignant tumor cells. In vivo functional assays revealed that CRNDE-induced tumor cells supported HCC progression, recruited abundant granulocyte myeloid-derived suppressor cells (G-MDSCs) and restricted the infiltration of T cells. In terms of mechanisms, CRNDE bound with Toll-like receptor 3 (TLR3) and activated NF-κB signaling to increase the secretion of c-x-c motif chemokine ligand 3 (CXCL3). CRNDE knockdown could significantly suppress the accumulation of G-MDSCs and enhance the infiltration of T cells in the TME of HCC in vivo. Taken together, our study reveals the CRNDE-NF-κB-CXCL3 axis plays a crucial role in driving the immunosuppressive niche to facilitate HCC progression by recruiting G-MDSCs.

## Introduction

Hepatocellular carcinoma (HCC) represents one of the most common fatal tumors, causing hundreds of thousands of deaths annually worldwide^1^. Prolonging the survival benefits of patients with unresectable advanced HCC has been an unsolved problem. Although multikinase inhibitors^2^, ^3^ and immune checkpoint inhibitors^4^-^6^ achieve promising outcomes in the treatment of advanced HCC, high levels of interpatient heterogeneity leave few patients with sensitive responses^7^, implying the complexity of mechanisms that facilitate HCC progression and emphasizing the urgency to identify novel mechanisms for optimal therapeutic options.

Although somatic mutations have been revealed to be central factors driving tumorigenesis^8^, numerous aspects of the tumor microenvironment (TME), including immune cell types, distribution density, location, and gain or loss of function, are considered crucial parameters associated with tumor progression, metastasis, prognosis and immunotherapy resistance^9^, ^10^. HCC tissues are typically characterized by abundant blood sinuses and lack stromata composed of miscellaneous cell types, such as fibroblasts (CAFs)^11^, infiltrating immune cells^12^, endothelial cells (ECs)^13^, and extracellular components that cause a complex TME to facilitate tumor progression. However, our understanding of the mechanisms by which TME-resident cell types drive HCC phenotypes and clinical trajectories remains nascent. Therefore, an increased exploration of the crosstalk between HCC cells and other TME-resident cells can impel the development of new therapeutic approaches for treating HCC. Myeloid-derived suppressor cells (MDSCs), which are naive myeloid cells resulting from bone marrow progenitor cells, are widespread in most types of tumors and recognized as predominant populations in restraining T-cell function^14^, ^15^. MDSCs are a heterogeneous population of immune cells identified in both mice and humans and are classified into granulocyte or polymorphonuclear MDSCs (G-MDSCs or PMN-MDSCs) and monocytic MDSCs (M-MDSCs)^16^, ^17^. MDSCs target T cells, B cells and NK cells to shape an immunosuppressive TME and hamper the effectiveness of cancer immunotherapy via the exhibition of immature morphology, weak phagocytosis, and the secretion of inhibitory cytokines such as chemokines, arginase-1 and reactive oxygen species (ROS)^18^. As the largest subfamily of micromolecule cytokines, chemokines can be released by various TME-resident cells and possess the ability to regulate oriented cell motion, adhesion, location, and cell‒cell communications. Chemokines not only recruit antineoplastic immune cells and maintain their functionality but also recruit immunosuppressive cells and inhibit the functionality of antineoplastic immune cells. CXCL1 and CCL5^19^ have been shown to recruit conventional type 1 dendritic cells (cDC1 cells) for the activation of antitumor immune cells; similarly, CXCL9 and CXCL10 have been reported to recruit CD8+ effector T cells (CD8+ Teff cells) to shape a tumor-immunoactivated TME 20. In contrast, tumor-associated macrophages (TAMs) recruited by CCL2^21^ and MDSCs recruited by CXCL1/CXCL3^22^ have been shown to be closely related to the immunosuppressive TME. Therefore, chemokines can be probed as therapeutic targets to improve the response to immunological therapies.

Colorectal tumor differential expression (*CRNDE*), which is affiliated with the long-chain noncoding RNA (lncRNA) class, was first found to be ectopic in colorectal cancer^23^ and subsequently verified to support the progression of various tumors involved in multiple myeloma^24^, glioma^25^ and HCC^26^. *CRNDE* has also been reported to modulate the levels of target genes through numerous approaches, including transcriptional regulation, posttranscriptional regulation, and competition for microRNA (miRNA) binding^27^. In addition, *CRNDE* has not only been confirmed to be closely connected with the proliferation, invasion and metastasis of HCC but also found to be in a position to cause radiation resistance^28^ and drug resistance to sorafenib^29^. However, the mechanisms by which ectopic *CRNDE* is linked to the TME in HCC have not been elucidated.

Here, we revealed that highly expressed *CRNDE* in mouse HCC cells significantly induced the release of CXCL3, which is critical for the recruitment of MDSCs to the TME of HCC. Very interestingly, we identified that *CRNDE* mediated the degradation of TLR3, which was essential for inducing the phosphorylation of NF-κB and nuclear translocation. These novel findings will be advantageous for understanding the molecular mechanisms involved in immunologic escape and probe into strategies targeting MDSCs and enhance response of HCC to immunotherapy.

## Materials and Methods

### Cell clusters

Human HCC cells HCCLM3 was purchased from American Type Culture Collection (Manassas, VA), Hep3B was purchased from Accgen (USA), mouse HCC cell Hepa1-6 was purchased from Procell, China, cultured in Dulbecco's Modified Eagle's Medium (DME) (Sigma-Aldrich) with 10% fetal bovine serum (FBS) (Sigma-Aldrich), and 1% Penicillin-Streptomycin (HyClone).

### Plasmids and lentivirus

*pX330-p53*(#59910), *pT3-EF1A-MYC-IRES-luc* (#129775) were purchased from Addgene (USA), *Crnde* overexpression plasmid for injection was restructured by *pT3-EF1A-MYC-IRES-luc*, *PCDH-CMV-MVS-EF1-Puro*, accomplished by GENERAL-BIOL (Anhui, China),* CMV-SB13* was gift from A. Lujambio (ISMMS, USA).* pCDH-CMV-MCS-3xFLAG-EF1-Puro*, *psPAX2*, and *pMD2.G* were gifts from Prof. Mian Wu (USTC). miR-384 mimic and si-RELA purchased from RiboBio (Guangdong, China).

Lentiviral packaging was performed by PEI mediated cotransfection of HEK293T cells with plasmids, collect supernatant after 48 hours and filter, HCC cells were transfected in 1600-800 µl fresh medium with 800-1600µl Lentiviral and 2-5µg/ml polybrene (Sigma, USA), change the medium after 12-24 hours, selected with puromycin (Sangon, Shanghai).

### Animals

4-5-week-old male C57BL/6 mice, weighing 16-20 g, all used in this experiment were purchased from Hangzhou Ziyuan Laboratory Animal Technology (Hangzhou, China). 20ug *pX330-p53*, 20ug *pT3-EF1A-MYC-IRES-luc*, 5ug PT2/C-Luc//PGK-SB13, with or not 20ug *Crnde*, dissolved in 2ml of 0.9% NaCl solution and inject 10% volume of mice body weight via tail vein within 3-5 seconds, generated mice HCC spontaneous tumor model, observed by bioluminescence imaging via IVIS software. The subcutaneous HCC tumor models were generated by inoculate Hepa1-6 cells into the mouse flank (75µl PBS 75µl Matrigel, 1×10^5^ cells per mouse). Tumors are measured daily, V= a×b^2^×π/6, a=maximum diameter, b=minimum diameter. Mice were treated with anti-PD1 or IgG 50 μg/20g, combined weit si-Crnde or si-Con 3nmol/20g. Ethical approval of laboratory animals was obtained from The First Affiliated Hospital of USTC Research Ethics Committee (2022-N(A)-046).

### Single-Cell Sequencing

The subcutaneous tumor tissue was taken out, stored in tissue protection solution and transported at 4°C. The tumor tissue was cut with scissors in 2.5ml cell culture medium with 10% FBS, and the interstitial tissue was removed by adding trypsin, collagens, etc. The cells were left to fall off and disperse at room temperature for 30-45min. The cells were filtered with a 70-micron cell filter (Jingan Biotechnology, Shanghai). Centrifuged at 700g for 10min, added appropriate amount of erythrocyte lysate, resuspended the cells, rested at 4°C for about 15 minutes, centrifuged at 700g for 5 minutes, removed the supernatant, added the cell fixing solution, resuspended the cells, and the single-cell suspension was configured. The gel beads containing barcode information were combined with the mixture of cells and enzymes to form GEMs (Gel Beads-In-Emulsions), reversed cDNA fragments were recorded, PCR amplification was performed using cDNA as a template, and a standard sequencing library was constructed. High-throughput sequencing was performed on the constructed library using the paired-end sequencing mode of the Illumina sequencing platform. Cell Ranger software was used to process the raw data to obtain each cell The gene expression matrix was further subjected to dimensionality reduction analysis using Seurat software to obtain UMAP visualization results, and all steps were completed by Lianchuan Biology (Hangzhou, China). Bioinformatic analysis was performed using the OmicStudio tools at https://www.omicstudio.cn/tool.

### RNA extraction and PCR

Total RNA was extracted using Gene JET RNA Purification Kit (Thermo Scientific™, USA), nuclear and cytoplasmic RNA was isolated with PARIS™ Kit (Invitrogen, USA), reverse transcribed into cDNA with PrimeScript™ RT Master Mix (TaKaRa, Japan), TB Green Premix Ex TaqTM II (TaKaRa, Japan) was used for qPCR, GAPDH and H1 were used as internal controls, and the standard 2-ΔΔCt method was used for calculation. All sequences of primers are listed in Supplementary Table 1.

### ELISA

Count 200,000-300,000 cells in 6-well plates, culture for 24-24 hours, collect the supernatant cell culture medium, and use CXCL1 ELISA KIT (Elabscience, China) and CXCL3 ELISA KIT (J&L Biological, China) to detect secretion in cell culture medium Concentrations of CXCL1 and CXCL3.

### RNA fluorescence in situ hybridization (FISH)

The RNA FISH kits were purchased from GenePharma (Shanghai, China). The paraffin sections or cell slides were processed according to the instructions. The RNA probes were hybridized with FAM (green) or CY3 (red) overnight at 37°C, and the nuclei were stained with DAPI. Filming was done within 2 days using a fluorescence microscope (Imager. M2, Zeiss). Tissue samples were obtained from patients with hepatobiliary surgery who agreed to participate in the research at The First Affiliated Hospital of USTC (Hefei, China).

### RNA pull-down assay

Sense and antisense strand probes 5' Biotin modification, synthesized by Sangon Biotech (Shanghai, China) and GENERAL BIOL (Anhui, China), Probe sequences are shown in Supplementary Table 1. Dynabeads M-280 Streptavidin (Invitrogen, USA) and probes mixed with cell lysate for 4 hours, collected the mixture for WB.

### RIP

There was no TLR3 antibody that can be used for RIP, so at first we constructed FLAG-tagged *TLR3* overexpression plasmid, transiently transfected into HCC cells, and then lysed with RIP lysis buffer. Flag antibody or IgG antibody was incubated with cell lysate at 4°C overnight, and then incubated with streptavidin dynabeads for 2 h, qPCR after RNA extraction.

### Flow cytometry

Fresh tumor tissues were removed, use scissors to shred it in sterile enzyme-free EP tube and put it into tube C, add 5mg enzyme A, 80mg enzyme D, 20mg enzyme R (Miltenyi Biotec, Germany), and add DMEM to 2.5ml, dissociated using gentleMACS™ Octo Dissociator (Miltenyi Biotec, Germany) for approximately 40 minutes, cells were filtered through 40 mm mesh, centrifuged at 700g for 7 minutes, and lysed by erythrocyte lysate (Biosharp, China) for 10-15 minutes , count 1 million in each group, T cells were stained with CD45, CD3, G-MDSCs were stained with CD45, CD11b, Ly6g, all flow antibodies in this paper are shown in Supplementary Table 2.

### Immunofluorescence (IF) and immunohistochemistry (IHC)

For IF, count 20,000 to 30,000 cell slides seeded in 24-well plates, fix with 4% paraformaldehyde (Biosharp, China) after 12-24 hours, 0.5% Triton X-100 permeabilization, blocking solution After blocking, the primary antibody was added at 4°C overnight, and the fluorescent secondary antibody was added the next day. After DAPI staining, the slides were mounted and stored in the dark, and photography was completed within 2 days. For IHC, paraffin sections were deparaffinized and rehydrated, antigen unmasking (VECTOR, USA), immersed in 3% hydrogen peroxide solution for 10 minutes, blocked with primary antibody overnight at 4°C, and then biotinylated secondary antibody (VECTOR, USA) and streptavidin HRP (VECTOR, USA), hematoxylin stained nuclues. All antibodies can be found in Supplementary Table 2.

### Statistical Analysis

Graphpad Prism 8 was used for statistical analysis. Student's T test was used for direct measurement results. P<0.05 represents a significant difference.

## Results

### Aberrantly expressed *CRNDE* is mainly manifested in malignant liver tumor cells

**Figure 1:**
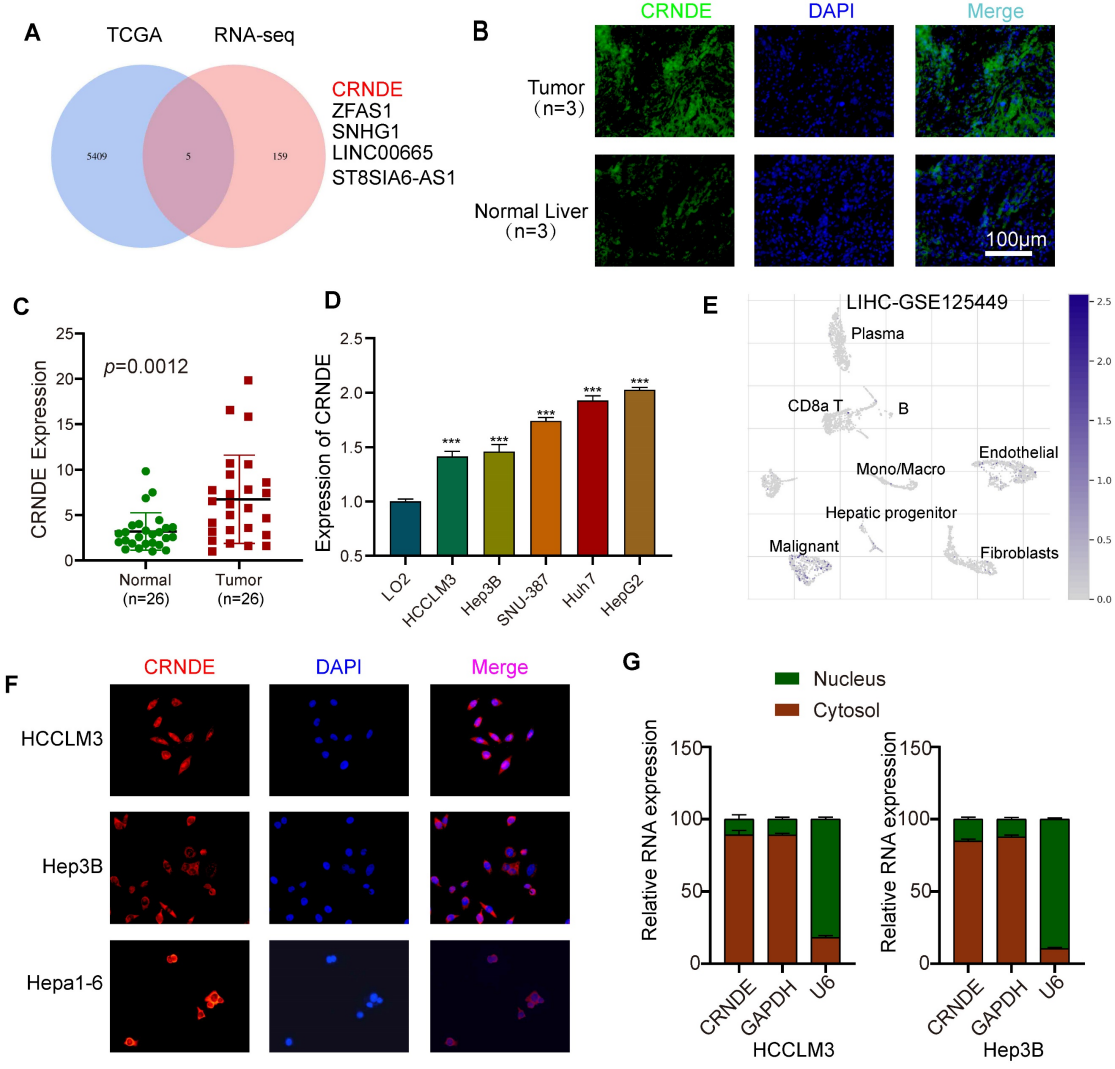
***CRNDE* is highly expressed in HCC and is mainly distributed in the cytoplasm of tumor cells. (A)** Venn diagram filtering 5 shared lncRNAs among the upregulated genes in TCGA database and overexpressed lncRNAs in tissue RNA sequencing results. **(B)** Representative images of FISH of *CRNDE* (green) in HCC tumors and the corresponding normal liver. Scale bar, 100 μm,n=3. **(C)** qPCR analysis of the expression level of *CRNDE* in clinical HCC tissue specimens (HCC tissue cDNA chip), n=26. **(D)**The expression of CRNDE in HCC cell lines. **(E)** The expression profile of *CRNDE* is shown in the UMAP from GSE125449. **(F)** The location of *CRNDE* (red) in HCClM3, Hep3B and Hepa1-6 cells was shown by FISH assay. Nuclei were stained with DAPI (blue); scale bar, 100 μm. **(G)** Analysis of the distribution of *CRNDE* in the nucleus/cytoplasm of HCCLM3 and Hep3b cells by qPCR. *GAPDH* was used as a cytoplasmic marker, and U6 was used as a nuclear marker.

To determine the target lncRNAs to be further studied, 5 candidate lncRNAs were screened out via combined analyses between the upregulated HCC gene sets from The Cancer Genome Atlas (TCGA) database and upregulated lncRNA datasets from transcriptome RNA sequencing (RNA-seq) involving 5 pairs of HCC and paracancerous tissues (Figure 1A). Given that CRNDE was the most significantly expressed in HCC, we regarded it as the target gene of interest for further research (Figure S1A, B). In line with previously published studies, the tissue in situ hybridization assay (FISH) showed that the fluorescence intensity of *CRNDE* in human HCC tissues was stronger than that in paired adjacent tissues, indicating that *CRNDE* was highly expressed in HCC (Figure 1B). Consistent results were observed in *CRNDE* RNA level assays of HCC tissue (Figure 1C). Compared with human normal liver cells (LO2), CRNDE was highly expressed in five HCC cell lines (Figure 1D). Subsequently, by interrogating the TISCH single-cell public database, we further confirmed that *CRNDE* is predominantly expressed in hepatic malignancies among different cell clusters in HCC tissues (Figure 1E). Likewise, this result is consistent with the single-cell RNA sequencing (scRNA-seq) results of C57BL/6 mouse subcutaneous tumors established with *Crnde*-mediated Hepa1-6 cells (Figure S1C). Furthermore, FISH assays revealed that *CRNDE* was generally localized in the cytoplasm (Figure 1F), which was consistent with the results of cytoplasmic and nuclear RNA fractionation (Figure 1G). These results suggest that *CRNDE* is highly expressed in HCC and distributed in malignant liver tumor cells, prompting us to examine the precise influence of *CRNDE* in HCC.

### *CRNDE* facilitates the development of HCC *in vivo*

**Figure 2:**
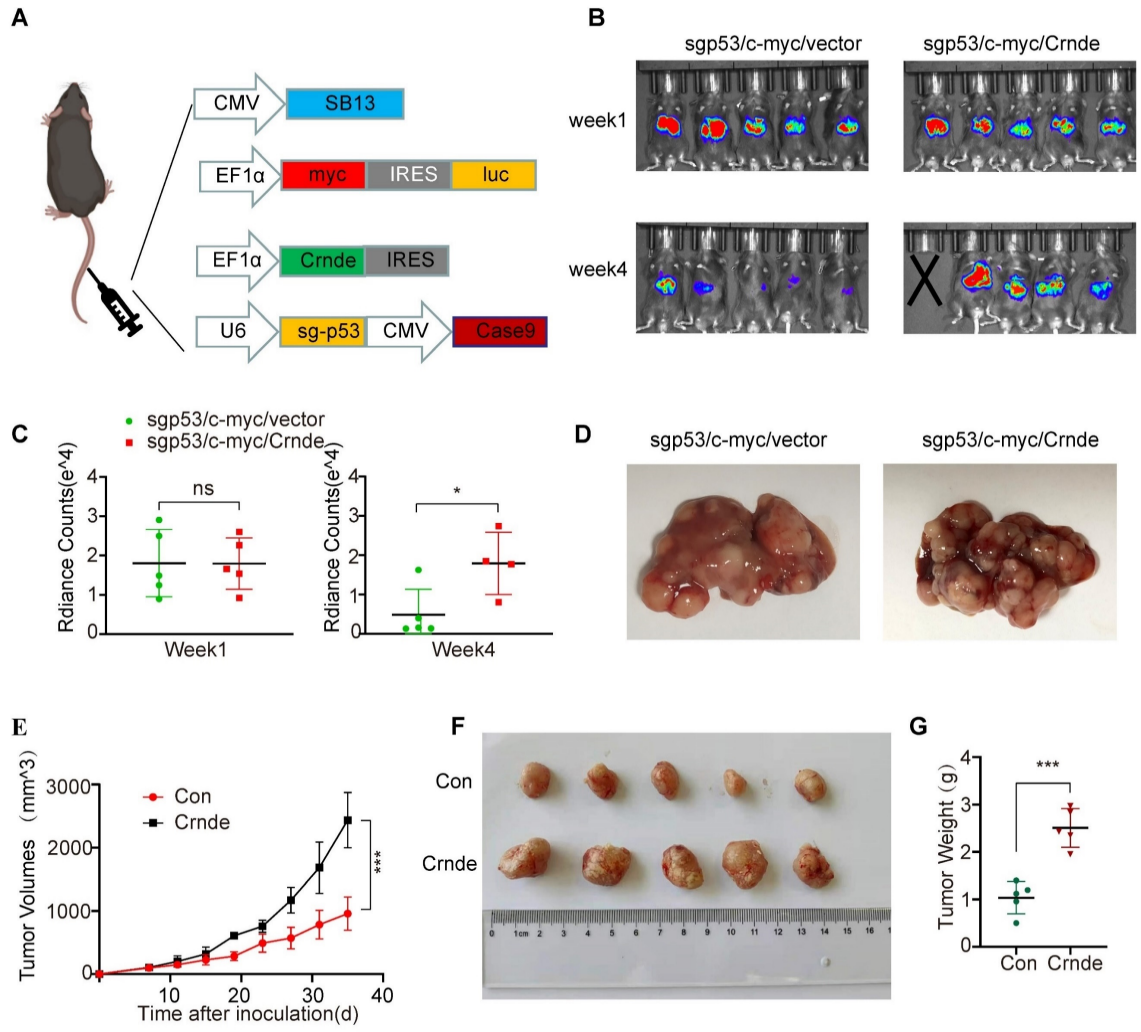
***CRNDE* promotes HCC progression *in vivo***. **(A)** Schematic of the plasmid used for hydrodynamic tail vein injection. **(B, C)** Bioluminescence imaging after injection of plasmids for 1 week and 4 weeks. Luciferase signals were quantified by using IVIS, n=5/group at the start of the experiment. **(D)** Representative images of spontaneous liver tumors isolated from two groups of C57/BL6 mice. **(E)** Growth curve of subcutaneous tumors in C57/BL6 mice after implantation of Hepa 1-6 cells stably transfected with the *Crnde*-overexpressing vector or control vector, n=5/group. **(F, G)** Mice were sacrificed, and subcutaneous tumors were collected. Images show volume and weight differences between the two groups. *p < 0.05, ***p < 0.001, ns indicates no significance.

To ascertain the characteristics of *CRNDE*-induced HCC *in vivo*, murine spontaneous HCC was caused by hydrodynamic tail vein injection of the combination of plasmids with upregulated *Crnde* or empty vectors and plasmids expressing *c-myc* and plasmids knocking out *p53* plus the transposase *SB13* in C57BL/6J mice (Figure 2A). The Crnde overexpression efficiency was confirmed based on the results of FISH assays of spontaneous mouse tumors (Figure S2A), and we cofirmed that the he tissues of the mice spontaneous tumor model have the pathological characteristics of HCC by IHC and HE staining (Figure S2B). Subsequently, the volume of liver-burdened tumors in mice was evaluated using the bioluminescence imaging system. The results revealed that the intensity of bioluminescence in sg*p53*/*c-myc*/*Crnde* mice was in line with that in sg*p53*/*c-myc*/vector mice one week after injection. However, sg*p53*/*c-myc*/*Crnde* mice exhibited more obvious bioluminescence intensity, larger tumor volume and more increased tumor numbers with the development of spontaneous tumors four weeks after injection (Figure 2B-D), implying that upregulated *Crnde* facilitated the development of HCC. Meanwhile, we subcutaneously injecting Hepa1-6 cells transfected with Crnde-overexpressing lentivirus (Figure S2C), established HCC subcutaneous tumor model and verified it has features of HCC (Figure S2D). We observed that the tumor proliferation rate, tumor volume and tumor weight were significantly increased in the *Crnde*-overexpressing group compared with the control group (Figure 2E-G), indicating that *Crnde* possessed tumor-proliferating properties. Thus, *CRNDE* might have potential for HCC development; however, whether *CRNDE* could efficiently influence the TME in HCC still needs further investigation.

### Single cell sequencing revealed the effect of *CRNDE* on HCC immune landscape

**Figure 3:**
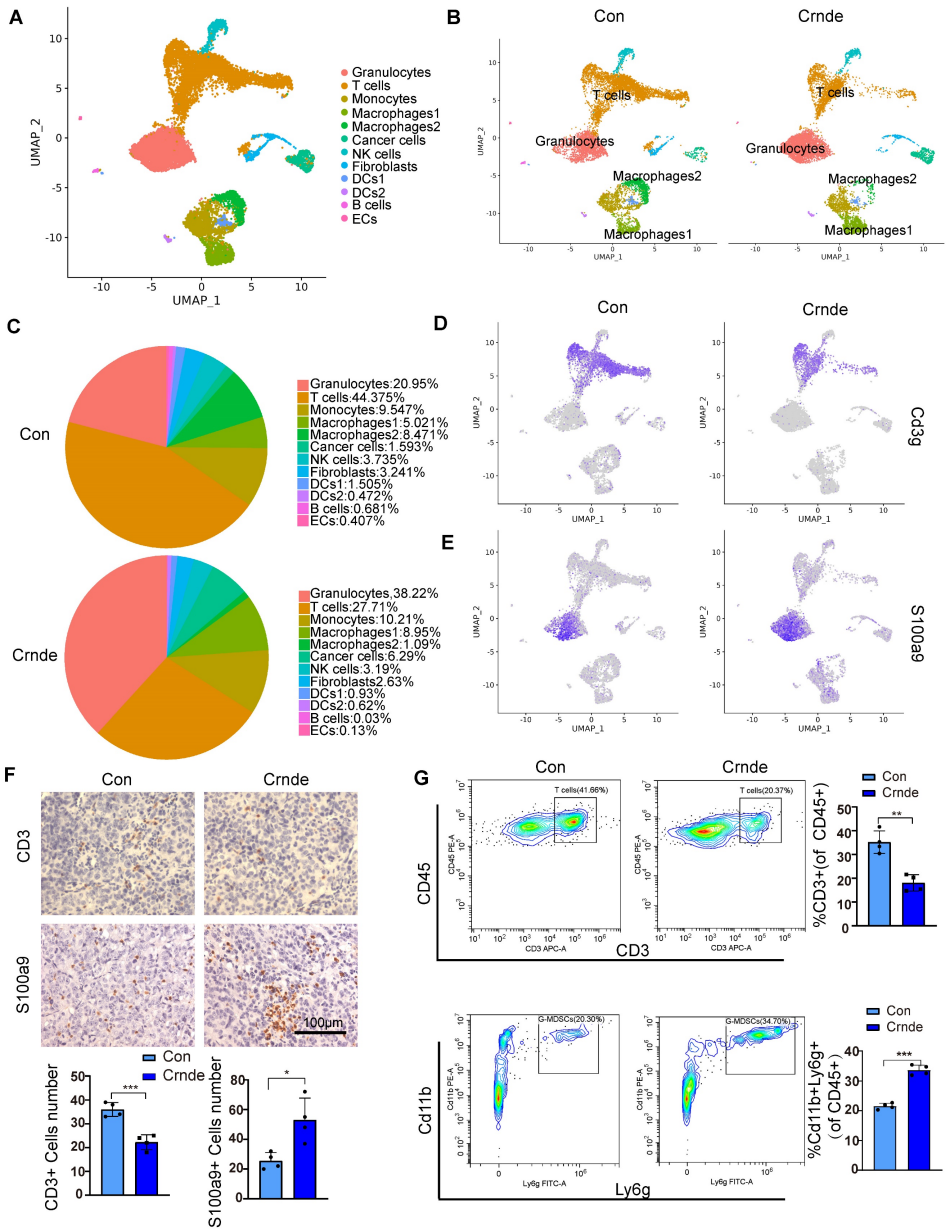
***CRNDE* promoted the recruitment of G-MDSCs to tumors and inhibited T-cell accumulation**. **(A)** The identification results of cell clusters collected from Con and *Crnde* mice subcutaneous tumors are shown in UMAP plots. **(B, C)** UMAP plots and pie chart plots were used to show differences in cell cluster distribution between the Con and *Crnde* groups.** (D, E)** Normalized expression levels of marker genes of T cells and G-MSDCs shown in the UMAP plot.** (F)** Representative IHC images, quantification of CD3 for T cells, S100a9 for G-MDSCs (n=4/group). Scale bar, 100 μm. **(G)** Flow cytometry was used to detect the proportion of T cells and G-MDSCs. CD45+CD3+ cells were T cells, and CD45+Cd11b+Ly6 g+ cells were G-MDSCs (n=4/group). Student's t test was used. *p < 0.05, **p < 0.01, ***p < 0.001.

To further explore whether *CRNDE*-induced HCC cells reshaped the TME *in vivo*, scRNA-seq (10X Genomics) was performed on the subcutaneous tumors of the above *Crnde* overexpression groups and the control groups, including a total of 17032 single cells (9,112 from the control groups and 7,920 from the* Crnde* groups) in the final datasets after quality control. Using cell type-specific canonical markers, the cell clusters were categorized into ten major cell types, referred to as granulocytes, T cells, monocytes, macrophages, cancer cells, NK cells, fibroblasts, dendritic cells (DCs), B cells and endothelial cells (ECs) (Figure 3A and Figure S3A). The most significant differences were observed in T cells and granulocytes of the *Crnde* overexpression groups compared with those of the control groups (Figure 3B, C). Overexpressed *Crnde* tumors showed significantly more decreased infiltration of T cells (Figure 3D). In addition, although granulocyte populations exhibited markedly increased accumulation in *Crnde*-overexpressing tumors, we found that *S100a9^high^/S100a8^high^* cells were highly enriched in the granulocyte populations (Figure 3E). Given that *S100a9* and *S100a8* have been reported to be important markers for the identification of G-MDSCs^30^, therefore, we believe that the differential granulocyte populations between the *Crnde* overexpression groups and the control groups included G-MDSCs. Consistent results were also observed in immunohistochemical staining and flow cytometry analyses of overexpressed *Crnde* and control tumors from mouse (Figure 3F, G), and the same results were found in tumor tissues of patients with HCC (Figure S4A, B). Although *Cd14*^31^, *Trem1*^32^, *Ptgs2*^33^ and *Cd274*^34^, ^35^ were upregulated in granulocytes, they have also been identified as markers of G-MDSCs(Figure S3B). However, *Elane*^36^, which encodes an important anticancer protein, was not expressed in granulocyte populations (Figure S3C). These results revealed that the majority of cells in the granulocyte populations were G-MDSCs.

In addition, we noticed macrophages 1 (characterized by immunosuppressive genes such ad *Arg1*,* Mrc1*,* Pf4*, *Trem2* and *Spp1*) was higher in *Crnde* overexpression group, in contrary, macrophages 2(characterized by proinflammatory genes such as *Cxcl9*, *Cd40*, *Cd74*, *H2-Ab1* and *H2- Aa*) was lower (Figure 3B, C and Figure S5A). To investigate whether changes of macrophage also play a role in Crnde mediated tumor growth promotion, we injected clodronate liposomes to delete macrophages (Figure S5B, C), the results did not show obvious difference (Figure S5D).

### G*-*MDSCs recruited by *CRNDE* overexpressed HCC cells restrict T-cell accumulation and activity

**Figure 4:**
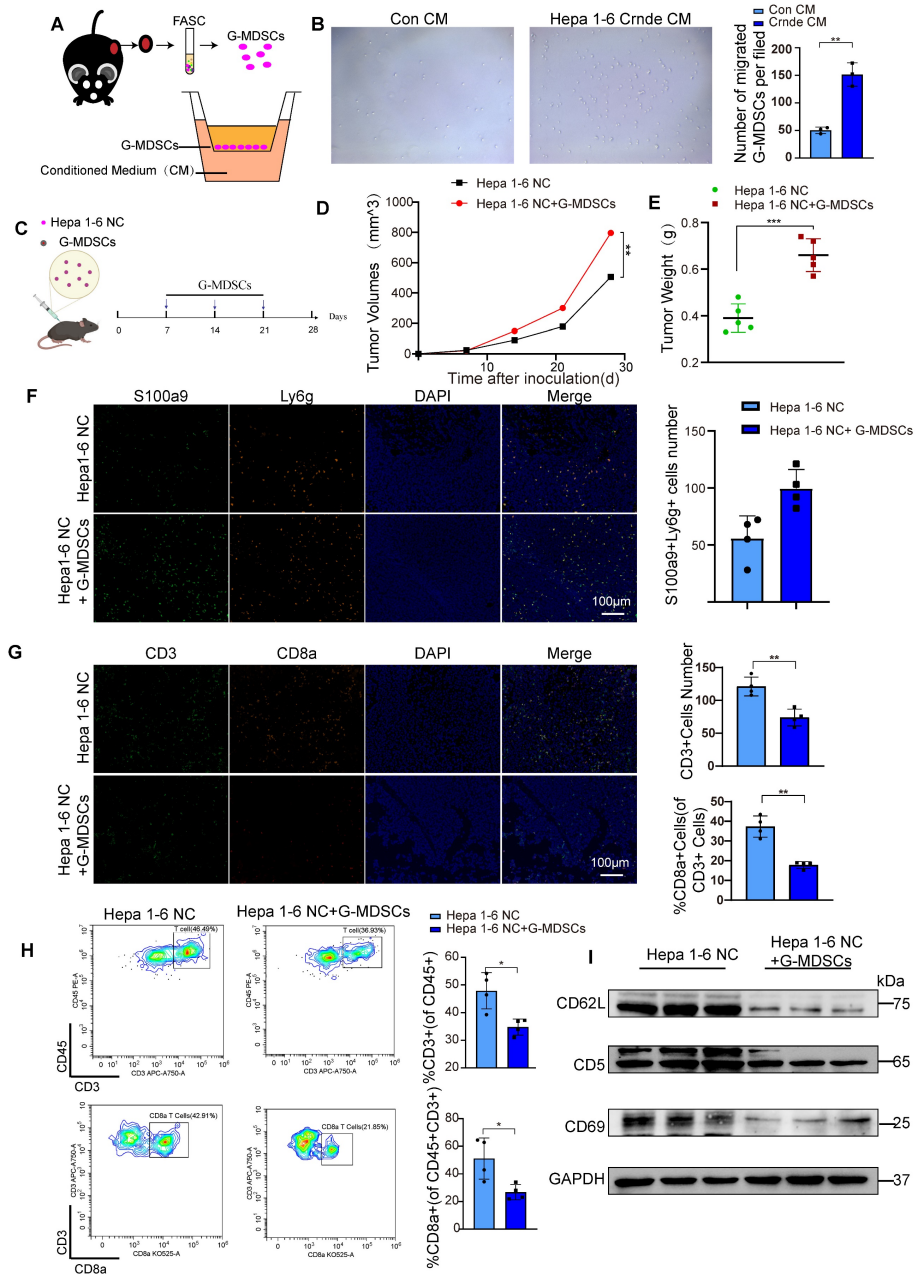
**G-MDSCs recruited by CRNDE overexpression in HCC cells inhibit T cell infiltration and activation. (A)** Schematic diagram of G-MDSCs transwell migration assay. **(B)** Total numbers of migrated G-MDSCs found in the lower chambers. **(C)** G-MDSCs were injected into a mouse subcutaneous tumor established by Hepa 1-6 NC cells. ** (D,E)** The tumor growth and tumor weight after inoculation (n=5 /group). **(F)** Multiple immunofluorescence images between two groups. green indicate S100a9, and orange indicate Ly6g, n=4 /group, scale bars, 100 μm.** (G)**Tissue sections were performed for multiplex staining, CD3 (green), CD8a (orange), n=4 /group, scale bars, 100 μm. **(H)** Flow cytometryanalysis of CD3+ T cells and CD8+ T cells in Hepa1-6 NC tumour-bearing mice treated with or without G-MDSCs(n=4/group).** (I)** Isolated T cells from two groups of tumor tissues, the expression of CD62L, CD5 and CD69 were examined by western blotting. *p < 0.05, **p < 0.01.

To further investigate whether the overexpression of CRNDE recruits G-MDSCs *in vitro*, we isolated G-MDSCs from subcutaneous tumors and seeded into transwell chamber, we placed the CM generated from Hepa1-6 Con and Hepa1-6 Crnde cells in the bottom wells (Figure 4A). After 24 hours of co-culture,we found Hepa1-6 Crnde CM significantly promoted the migration of G-GMDSCs(Figure 4B).We were also curious of these G-MDSCs recruited by CRNDE overexpression whether play an important role in HCC progression. So, we isolated G-MDSCs from Hepa1-6 Crnde group and implanted them into the subcutaneous tissue of mice together with Hepa1-6 NC cells, and injected G-MDSCs once a week (Figure 4C). Not surprisingly the injection of G-MDSCs strongly promoted the growth of HCC and increased the weight of tumor (Figure 4D, E). Firstly, we verified that ly6G+S100A9+ G-MDSCs were increased after injtected G-MDSCs (Figure 4F). Consistent with the results had been shown in single-cell sequencing, G-MDSCs recruited by Crnde overexpressed HCC cells inhibited the infiltration of T cells, especially the CD8+ T cells (Figure 4G, H and Figure S4C). In addition, homing and activation-related proteins L-selectin (CD62L), CD5 and CD69 in T cells were down-regulated to varying degrees in G-MDSCs group (Figure 4I), which suppressed the antitumor immune activity of tumor-infiltrating T cells.

Thus, these results indicate that CRNDE-induced HCC cells potently drive the immunosuppressive activity of MDSCs to restrict T-cell accumulation and activity in the TME.

### CXCL3, a potent MDSC chemoattractant, is secreted abundantly by *CRNDE*-treated HCC cells

**Figure 5:**
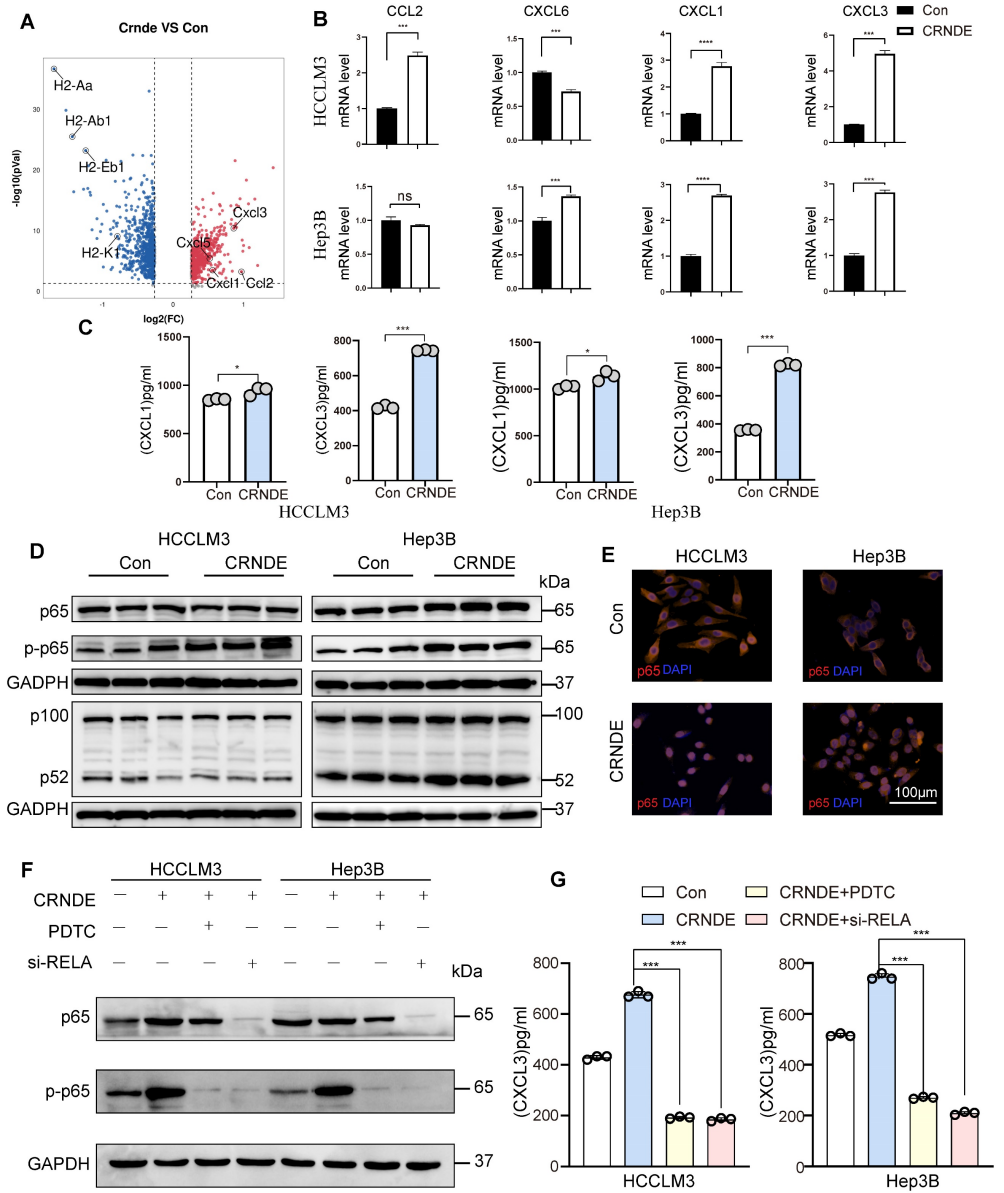
***CRNDE* promotes CXCL3 secretion through the NF-кB pathway**. **(A)** Volcano plot showing the differentially expressed genes in cancer cells between the two groups*, Ccxl1*. *Cxcl3*, *Cxcl5* and *Ccl2* were identified, -log2FC≥0.5 and *p*<0.05. **(B)** The mRNA levels of *CXCL1*, *CXCL3*, *CXCL6* and *CCL2* in HCCLM3 and Hep3B cells were analyzed by using qPCR. **(C)** CXCL1 and CXCL3 secreted by HCC cells were detected by ELISA. **(D)** NF-кB pathway-related proteins were detected by western blot in HCCLM3 and Hepa3B cells after overexpression of *CRNDE*. **(E)** Localization of the NF-кB p65 subunit in HCC cell lines is shown in the immunofluorescence image. p65 (orange) and nucleus (blue), scale bar, 100 μm. **(F)** Westren blotting assay of p65 and p-p65 in si-RELA or PDTC treated HCC cells.** (G)** ELISA assay of CXCL3 in si-RELA or PDTC treated HCC cells. **p < 0.01, ***p < 0.001, ns means no significance.

To elucidate the factors governing G-MDSC recruitment in *CRNDE*-induced HCC, we examined putative G-MDSC-recruiting chemokines, indicating that the expression of *Cxcl3*, *Cxcl1*, *Cxcl5*, and *Ccl2* was significantly increased in upregulated *Crnde* tumor cells (Figure 5A). Thereafter, we applied qRT‒PCR analyses to examine the mRNA levels of *CXCL3*, *CXCL1*, *CXCL6* (the human homolog of mouse *Cxcl5*) and *CCL2* in *CRNDE*-overexpressing HCCLM3 and Hep3B cells and found that only *CXCL1* and *CXCL3* were significantly elevated in both cell lines (Figure 5B), implying that *CXCL1* and *CXCL3* were major factors recruiting G-MDSCs in *CRNDE*-induced HCC. Moreover, ELISAs showed increased expression and presence in the culture medium of *CRNDE* HCCLM3 and Hep3B cells overexpressing *CXCL1* and *CXCL3*, with *CXCL3* showing a dramatic increase (Figure 5C). Besides, we also used TCGA and GETx database to analyze cytokines in HCC, and the result showed that CXCL3 was positively correlated with CRNDE (Figure S6A). Blocked Cxcl3 by anti-Cxcl3 effectively abrogated Crnde induced tumor growth (Figure S6B, S6C), and G-MDSCs migration in *vivo* and *vitro* (Figure S6D, S6E). Together, these findings demonstrate that *CRNDE*-mediated CXCL3 secretion is an important factor in promoting tumor growth and G-MDSCs recruitment.

### NF-κB is activated by CRNDE in HCC cells and regulates CXCL3 expression

To explore *CRNDE* regulation of *CXCL3* expression, NF-κB was speculated to be activated based on previous studies that identified NF-κB signaling as a crucial regulator of inflammation-related cytokines influenced by *CRNDE*^37^. Subsequently, we found that ectopic *CRNDE* had no effects on noncanonical pathways of NF-κB. However, it induced p65 phosphorylation (Figure 5D) and stimulated the nuclear translocation of p65 (Figure 5E), which was confirmed by western blot and immunofluorescence analyses in the canonical and noncanonical NF-κB pathways. To investigate the functional relevance between NF-κB and *CXCL3*, we used si-RELA to knockdown p65, and treated HCC cells with the NF-κB inhibitor pyrrolidinedithiocarbamate ammonium (PDTC), observed that the upregulation of *CXCL3* secretion levels induced by *CRNDE* was blocked (Figure 5F, G). Taken together, these findings reinforce the role of NF-κB in the regulation of CXCL3 expression and subsequent increased G-MDSC recruitment specifically in CRNDE-induced HCC.

### *CRNDE* interacts with TLR3 and inhibits its degradation

To further clarify the mechanism by which *CRNDE* activates NF-κB, Toll-like receptor 3 (TLR3) was given more attention in consideration of its functional correlations with NF-κB signaling^38^. Moreover, previous studies have revealed that *CRNDE* promotes sepsis-induced renal injury through the TLR3/NF-κB pathway^39^.

**Figure 6:**
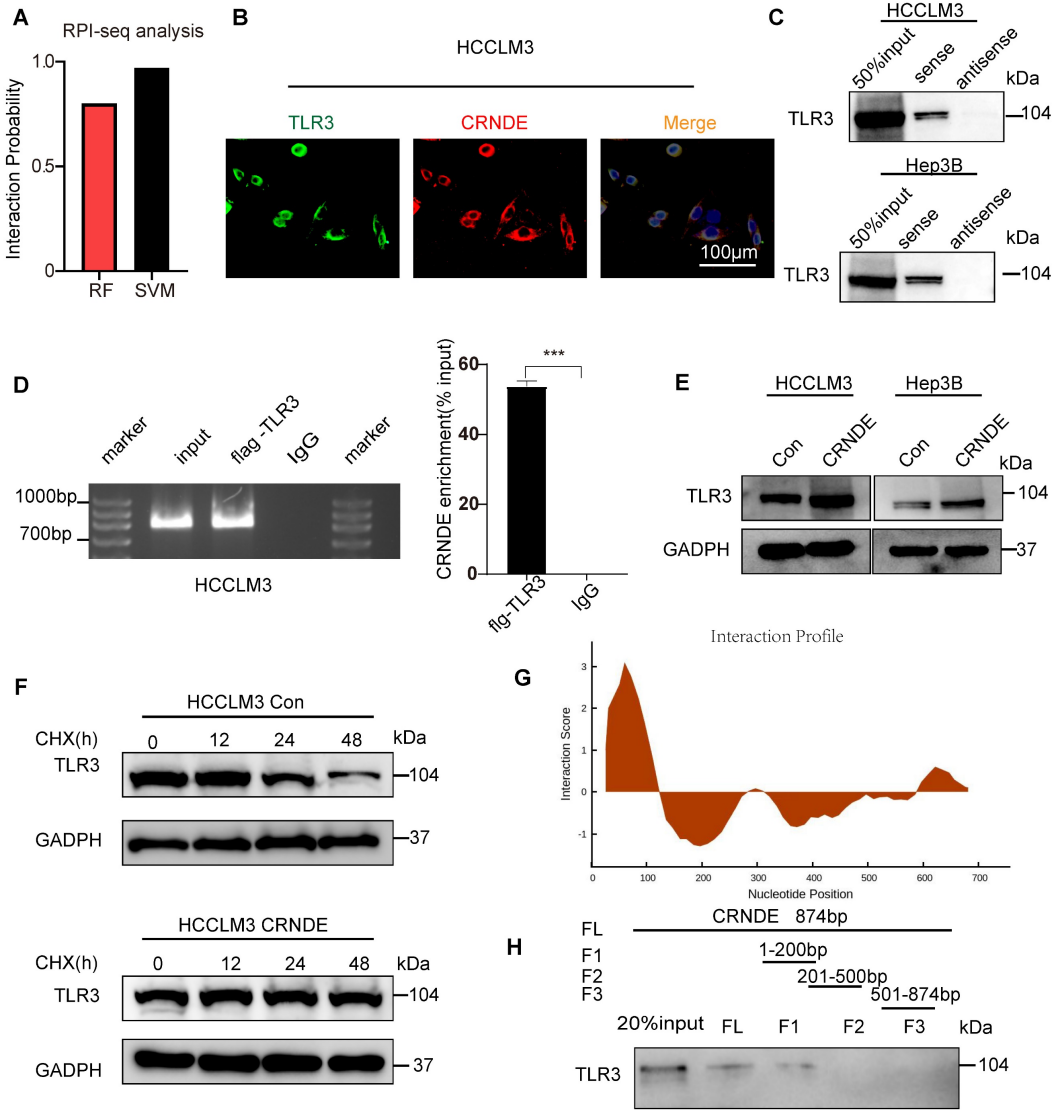
***CRNDE* binds to TLR3 and inhibits its degradation**. **(A)** RPI-seq platforms (http://pridb.gdcb.iastate.edu) strongly suggest that *CRNDE* interacts with TLR3. p > 0.5 was considered “positive”. (B) Colocalization of *CRNDE* (red) and TLR3 (green) in HCCLM3 cells is shown in FISH-IF images. **(C)** RNA pulldown assay showed that *CRNDE* interacted with TLR3 in HCCLM3 cells; scale bar, 100 μm. **(D)** RIP results also verified that *CRNDE* interacted with TLR3. **(E)** Western blot showing the levels of TLR3 after *CRNDE* overexpression in HCC cells. **(F)** A cycloheximide chase assay was used to observe the half-life of the TLR3 protein in *CRNDE*-overexpressing cells.** (G)** The sequence of *CRNDE* binding to TLR3 protein was predicted by the catRAPID platform (http://service. tartaglialab. com). **(H)** RNA pull-down results of biotinylated full-length; fragment 1 (1-200 bp); fragment 2 (201-500 bp); F3, fragment 3 (501-874 bp).

However, the mechanisms linking *CRNDE* and TLR3 in malignant tumors have not been elucidated. Intriguingly, using the RPI-seq prediction platform (http://pridb.gdcb.iastate.edu), we observed that there was a high possibility of an interaction between *CRNDE* and TLR3 (Figure 6A). To confirm the reliability of the results of the public database, we first applied the combination of FISH and immunofluorescence assays (FISH-IF) to detect the colocalization of *CRNDE* and TLR3 in the cytoplasm of HCC cells (Figure 6B and Figure S7A). Next, RNA pulldown was performed in HCC cells to demonstrate that TLR3 was only precipitated by sense *CRNDE* (Figure 6C and Figure S7B), indicating the existence of the interaction between *CRNDE* and TLR3. Consistent results were observed in RNA immunoprecipitation (RIP) assays (Figure 6D and Figure S8C); however, q-PCR analyses showed that there were no changes in *CRNDE* mRNA levels (Figure S8D). Since previous works have revealed that ectopic *CRNDE* can elevate the protein levels of TLR3 (Figure 6E), we hypothesized that TLR3 stability in HCC cells might be regulated by *CRNDE*. Using cycloheximide (CHX) to treat *CRNDE*-overexpressing and control HCC cells for various durations, we determined that the half-life of TLR3 was significantly prolonged in CRNDE-overexpressing HCC cells, indicating that *CRNDE* strengthened the stability of TLR3 and inhibited its degradation (Figure 6F). To ascertain the RNA binding domain essential for the *CRNDE*-TLR3 interaction, we generated several *CRNDE* mutants by deleting different domains (F1: delete 201-874 bp; F2: delete 1-200 bp and 501-874 bp; F3: delete 1-500 bp) based on the predicted binding sequences used by the catRAPID platform (http://service.tartaglialab.com). *In vitro* RNA‒protein binding assays showed that F1 mediated its interaction with TLR3, and F2 and F3 were not required for its interaction with TLR3 (Figure 6G, H). To further test whether deleting F1 had impacts on the expression levels of TLR3, p65 phosphorylation and the secretion of CXCL3, we constructed a *CRNDE* mutant with a deletion of 1-200 bp named *CRNDE* △F1. Then, Western blot and ELISAs were performed to verify that the expression of TLR3 and p65 phosphorylation were significantly downregulated by *CRNDE* △F1 compared with *CRNDE*-FL (Figure S7E), as was the secretion of CXCL3 (Figure S7F). Together, the phosphorylation of p65 and secretion of CXCL3 caused by *CRNDE* is dependent on the stability of TLR3 strengthened by the nucleotide-binding domain of *CRNDE*.

It also has been reported that CRNDE upregulates the expression of NF-κB by though inhibition of miR-384^40^, thus we wondered whether miR-384 suppress the secretion of CXCL3. In practice, although miR-384 inhibited p65 expression, it had no significant effect on p65 phosphorylation induced by CRNDE (Figure S8A), there was no significant inhibition of CXCL3 secretion either (Figure S8B).

### Target CRNDE enhances the efficacy of immunotherapy in HCC

**Figure 7:**
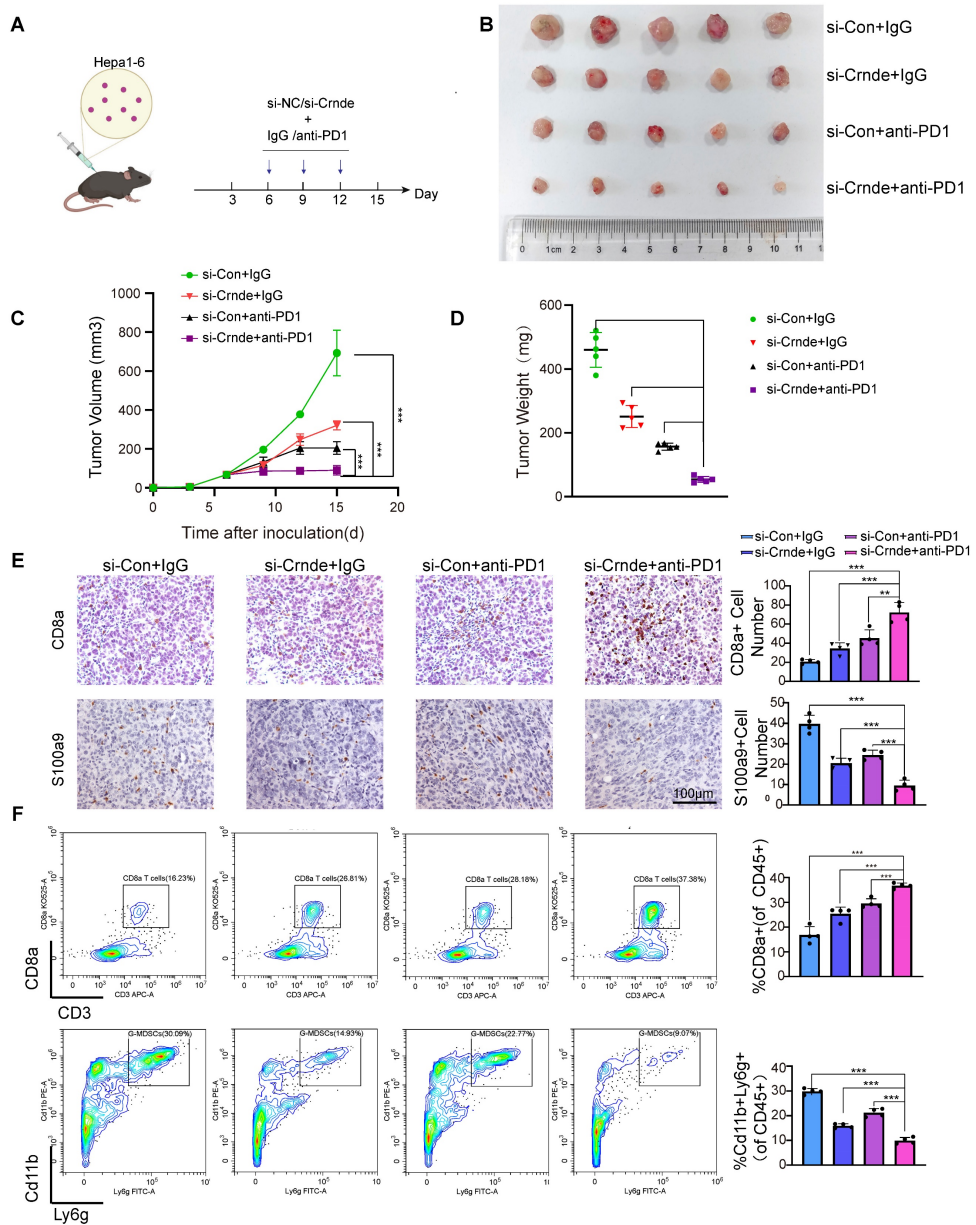
** Target CRNDE can enhance responses to immunotherapy in HCC. (A)** Flowchart of the HCC subcutaneous tumor model, anti-PD1 or IgG 50 μg/20g, si-Crnde or si-Con 3nmol/20g, n=5 per group. **(B)** The mice were sacrificed after 28 days, and the final subcutaneous tumor is shown.** (C, D)** Tumor growth and weight of mice in the 4 groups. **(E)** CD8+T cells and G-MDSCs were assessed using IHC, CD8a for CD8+T cells, S100a9 for G-MDSCs. n=4/group, scale bar, 100 μm. **(F)** CD8+T cells and G-MDSCs in each group were analyzed by flow cytometry. n=4/group, ***p < 0.001.

Considered that CRNDE can recruit G-MDSCs and inhibit the infiltration of T cells, promoted immune suppression in HCC, we were curious about whether targeted CRNDE can improve the efficacy of immunotherapy. Therefore, we treated subcutaneous tumors implanted by hepa 1-6 with si-Crnde(*in vivo*) and anti-PD1(Figure 7A). As expected, mice treated with combination of si-Crnde and anti-PD1 formed significantly smaller tumors (Figure 7B-D), with more increased infiltration of CD8+ T cells and less recruitment of G-MDSCs (Figure 7E, F), compared with individual treatments. These results revealed that targeting CRNDE has a great potential in immunotherapy of HCC.

## Discussion

With the advent of tumor immunotherapy, agents impacting the efficacy of tumor immunotherapy and the interplay across various TME-associated cells have received more attention. LncRNAs are not only regarded as regulators of biological processes of tumor cells, including proliferation, metastasis and metabolism but also have the capabilities to modulate the TME^41^. In our study, the combined evaluations between TCGA database and HCC tissue arrays revealed that *CRNDE* was screened out for candidate genes and was highly expressed in HCC. Accumulating evidence has shown that *CRNDE* can mediate the biological processes of various tumor cells. Moreover, previous studies have reported that *CRNDE* from colorectal cancer exosomes can promote Th17-cell differentiation by inhibiting RORγt degradation^42^, implying that *CRNDE* also regulates the activation and motion of immune cells in the TME. Nevertheless, both public single-cell databases and our scRNA-seq results of mouse subcutaneous tumors revealed that *CRNDE*-positive cells largely manifested in malignant tumor cell populations. Therefore, we further investigated the interplay between *CRNDE*-induced HCC cells and other TME-resident cells.

Granulocytes are one of the most common and abundant TME-resident cell types in HCC. Detailed explorations of the crosstalk and distribution of granulocytes in the TME have cataloged the diverse functions and types, which are increasingly considered potential sources of therapeutic targets. Mounting evidence also points to double-edged sword roles for granulocytes in the TME, involving the secretion of ELANE^36^ to restrict tumor progression and the polarization of Th cells to promote tumor progression^43^. Granulocytes facilitating tumor development in the TME were mainly due to G-MDSCs, which were abundantly accumulated in most tumors and tumor-bearing mice. In our study, we found that ectopic *CRNDE* not only facilitated HCC progression but also significantly affected the distribution of immune cells, among which the most prominent changes were an increased percentage of granulocytes (38.22% to 20.93%) and a decreased percentage of T cells (27.72% to 44.41%). Given that upregulated genes in granulocyte populations were markers of G-MDSCs, such as *S100a9*, *S100a8*, *Trem1* and *Cd14*, we believe that the differential granulocyte populations between the *CRNDE* overexpression groups and the control groups included G-MDSCs. In addition, previous studies revealed that COX-2 (encoded by *Ptgs2*) and PDL1 (encoded by *Cd274*) could restrict the proliferation and activation of T cells, implying that CRNDE-induced HCC cells not only increased the recruitment of G-MDSCs to impede the infiltration of T cells but also repressed the activation of T cells.

Various chemokines have been reported to drive the motion of G-MDSCs in the TME, including CXCL1, CXCL2, CXCL3 and CXCL8^44^. To explore the link between CRNDE and G-MDSCs, we observed that Crnde overexpression triggered the activation of *Cxcl3*, *Cxcl1*, *Cxcl5* and *Ccl2* in HCC cells. Via qPCR and ELISA analyses, CXCL3 was identified as the chemokine most closely related to *CRNDE* in the TME of HCC. Given that previous studies revealed that *CRNDE* could activate TLR3/NF-κB signaling to secrete numerous inflammatory factors, we hypothesized that *CRNDE* could affect the protein levels of NF-κB to secrete CXCL3 in HCC cells. However, our work showed that *CRNDE* increased the phosphorylation and nuclear translocation of p65 to promote the secretion of CXCL3 in HCC cells instead of affecting the protein levels of NF-κB. In addition, the mechanism by which *CRNDE* contributes to NF-κB activation has not been well investigated, and we determined that the 1-200 bp fragment of *CRNDE* could bind with TLR3 and strengthen the stability of TLR3 to activate NF-κB instead of affecting the protein levels of TLR3. Finally, to better understand the role of CRNDE in immunotherapy of HCC, therapeutic models were established, showed targeting CRNDE in combination with anti-PD1 has significant inhibitory effect on HCC growth.

In conclusion, our study demonstrates that the CRNDE-NF-κB-CXCL3 axis plays a crucial role in driving the immunosuppressive niche to facilitate HCC progression by recruiting G-MDSCs. Our study also provides a novel therapeutic strategy for HCC patients with highly expressed CRNDE.

## Supplementary Material

Supplementary figures and tables.
