# Supplementary material for: LncRNA CRNDE Drives the Progression of Hepatocellular Carcinoma by inducing the Immunosuppressive Niche
Source: Int J Biol Sci. 2024 Jan 1;20(2):718–32. doi: 10.7150/ijbs.85471 (PMC10758109; doi:10.7150/ijbs.85471)
Supplement: Supplementary file 1 — Supplementary figures and tables. [file ijbsv20p0718s1.pdf]

Supplementary Data:

Figure Legends

Figure S1. *CRNDE* is overexpression in HCC and it is hardly expressed in immune cells. (A) Volcano plots showing the significantly upregulated lncRNAs in tissue RNA sequencing. **(B)** TCGA data shows *CRNDE* is up-regulated in HCC. **(C)** Violin plots shown *Crnde* was mainly expressed in cancer cells in mice.

Figure S2. Characteristics of mice HCC models. (A) FISH was used to verify the *Crnde*(Green) over-expressed after injected plasmids. scale bar, 100 μm , n=3. **(C)** qPCR was used to notarize *Crnde* was upregulated in Hepa1-6 cells. **(B, D)** The mice spontaneous tumors and subcutaneous tumors were vertified by IHC and HE staining. scale bar, 200 μm, n=3.

Figure S3. Marker gene analysis of clusters concerned. (A) Bubble map of the marker genes in the identified cell subsets. **(B,C)** Visualization of MDSCs marker genes in UMAP plot.

Figure S4. CRNDE promotes the recruitment of G-MDSCs and reduces T cell infiltration in human HCC. (**A, B**) Representative pictures of CD3 and S100a9 IHC in High CRNDE and Low CRNDE expression tumor tissues of patients with HCC(n=10/group), Scale bars, 100 μm in 20× images (top rows) and 200 μm in 40× images (bottom rows). （**C**） Visualization of CD8a in UMAP plot. ***p < 0.001.

Figure S5. The alteration of macrophages had no significant effect on CRNDE-mediated tumor promotion. (A) Dot plots showing the expression of immunosuppressive and proinflammatory genes among macrophages1 and macrophages2. **(B,C)** Representative plots, percentages of macrophages from three groups ,n=4.**(D)** Growth curves of subcutaneous tumor, n=5. ***p < 0.001，ns means no significance.

Figure S6. CXCL3 plays an important role in tumor growth and recruitment of G-MDSCs in CRNDE overexpressed HCC. (A) Person analysis of cytokine, data fromTCGA&GTEx, analysed by using Sangerbox 3.0. **(B,C)** Tumor growth curve and weight, n=5. **(D)** Percentages of G-MDSCs *in vivo* were analysed by flow cytometry. **(E)** The number of migrated G-MDSCs were detected by migration assay in vitro,n=3.***p <

0.001.

Supplementary Figure S7. ***CRNDE* binds to TLR3 and affect TLR3 depend on fragment 1 (1–200bp)**. **(A)** Colocalization of *CRNDE* (red) and TLR3(green) in Hep3B. **(B)** RNA pull down combined with protein silver staining to detect CRNDE specific binding protein. **(C)** RIP results of Hep3B. **(D)** TLR3 mRNA levels were detected by PCR. **(E，F)** TLR3， p-p65 and CXCL3protein levels were detected after transfected with truncated fragment 1 in HCCLM3 and Hep3B. ***p < 0.001，ns means no significance

Figure S8. miR-384 had no significant effect on the phosphorylation of p65 and CXCL3 secretion. (A) Western blot analysis p65 and phosphorylation p65 protein levels in HCCLM3 and Hep3B cells treated by CRNDE and miR-384 minics.**(B)** Elisa analysis the secretion of CXCL3 treated HCCLM3 and Hep3B cells.

Figure S1

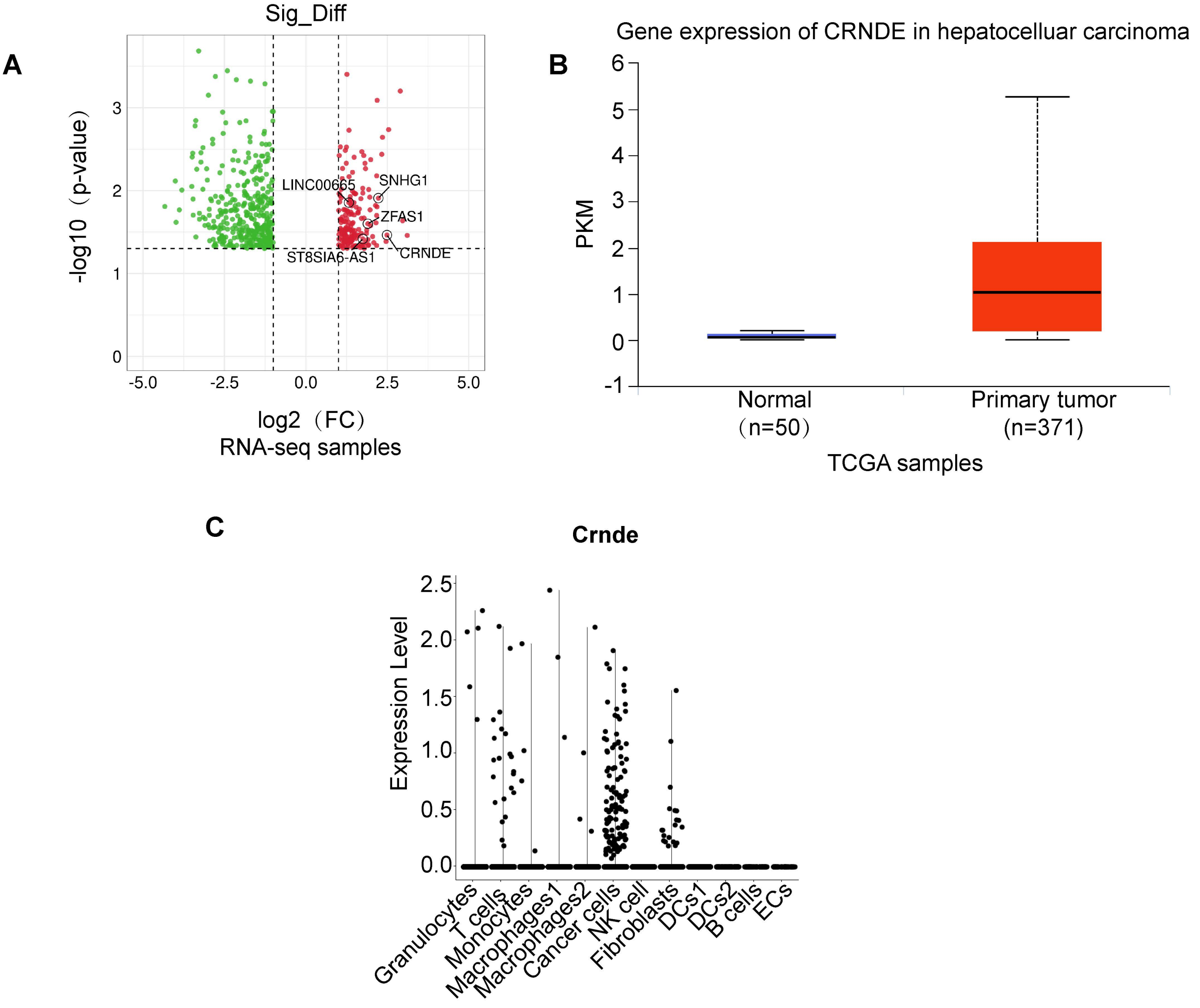
A
Sig_Diff
-log10（p-value）
log2（FC）
RNA-seq samples
LINC00665
SNHG1
ZFAS1
ST8SIA6-AS1
CRNDE
B
Gene expression of CRNDE in hepatocelluar carcinoma
PKM
Normal
（n=50）
Primary tumor
(n=371)
TCGA samples
C
Crnde
Expression Level
Granulocytes
T cells
Monocytes
Macrophages1
Macrophages2
Cancer cells
NK cell
Fibroblasts
DCs1
DCs2
B cells
ECs

Figure S2

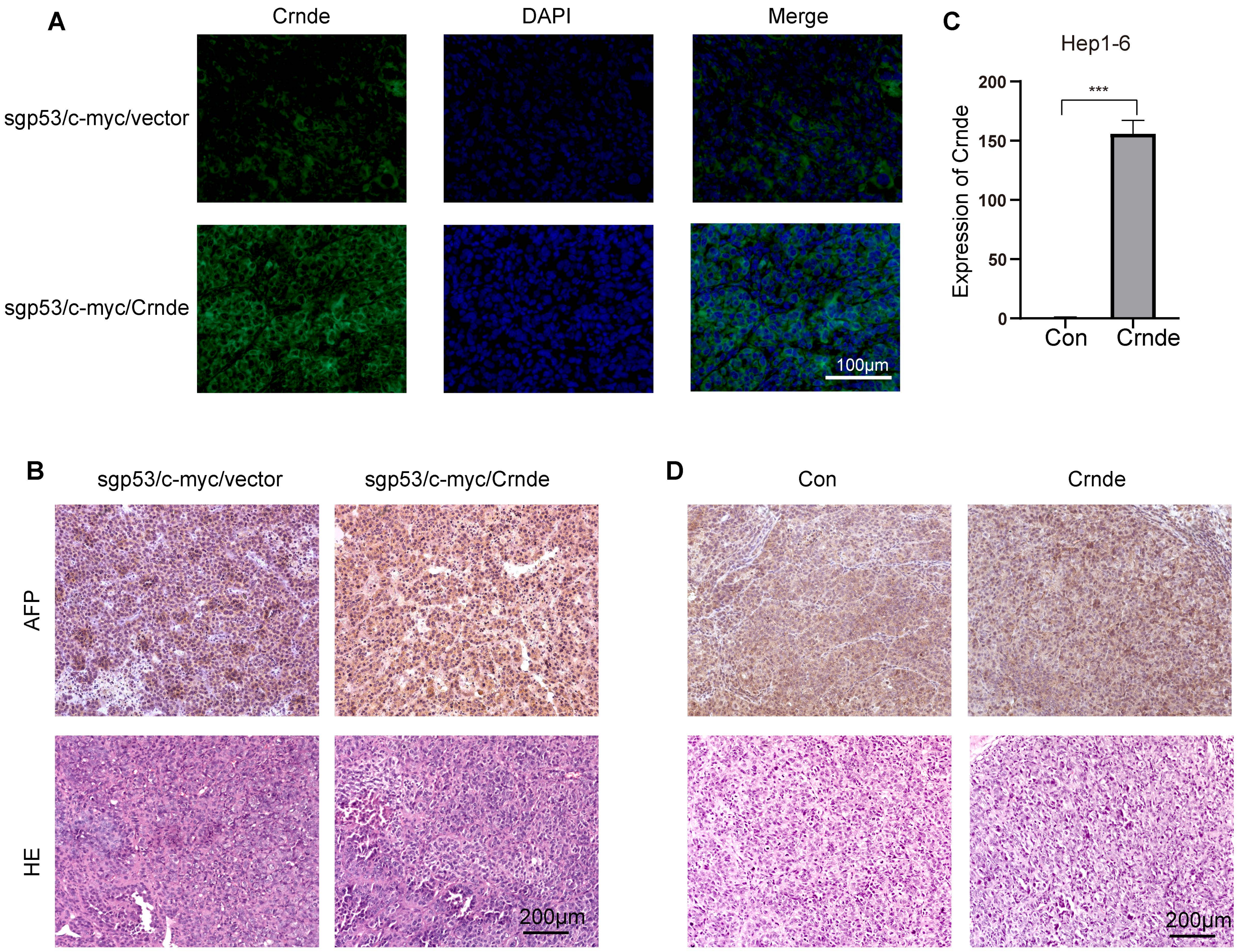

A
Crnde
DAPI
Merge
sgp53/c-myc/vector
sgp53/c-myc/Crnde
100μm
C
Hep1-6
Expression of Crnde
200
150
100
50
0
***
Con
Crnde
B
sgp53/c-myc/vector
sgp53/c-myc/Crnde
AFP
HE
200μm
D
Con
Crnde
200μm

Figure S3

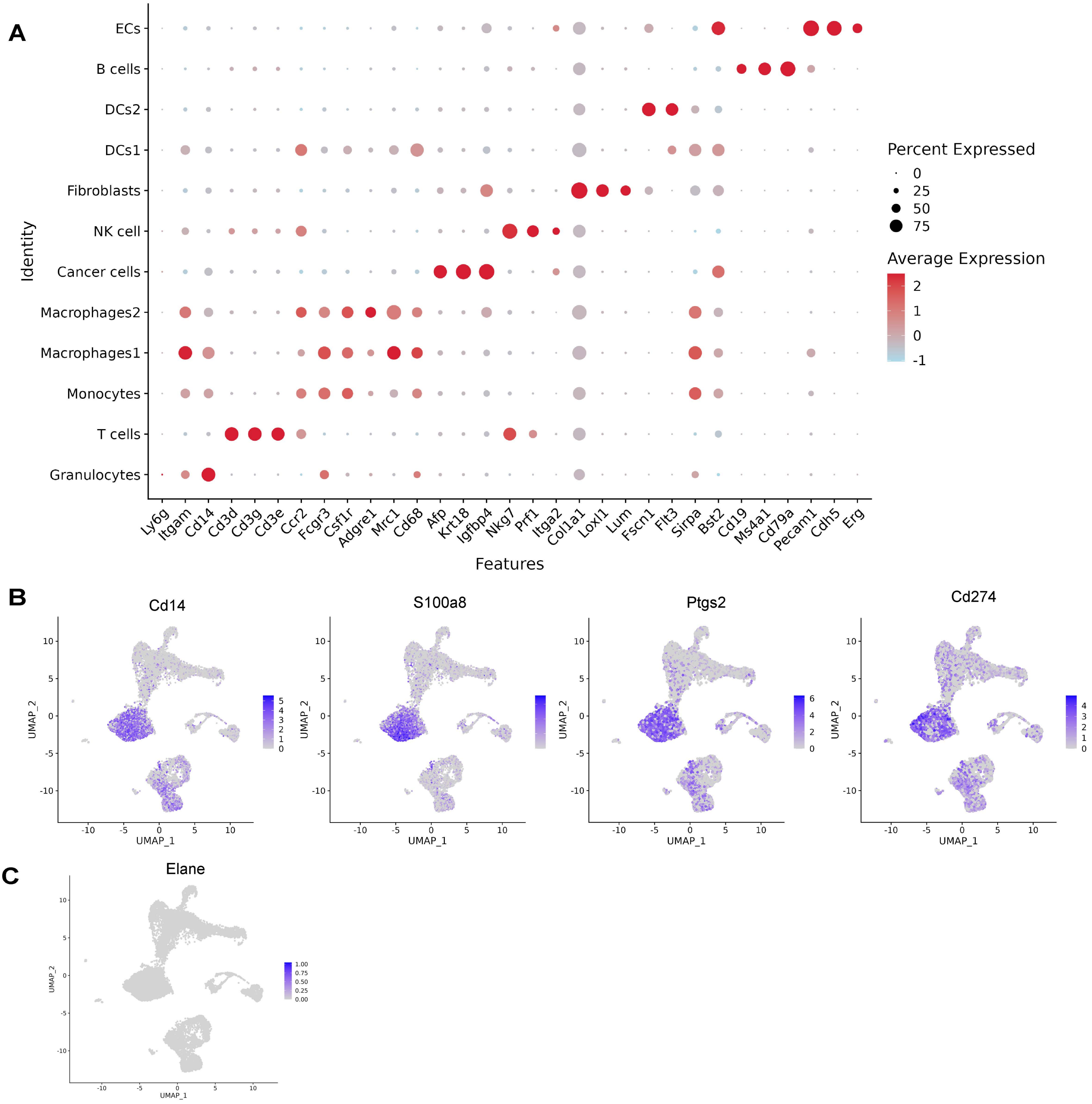
A
ECs
B cells
DCs2
DCs1
Fibroblasts
NK cell
Cancer cells
Macrophages2
Macrophages1
Monocytes
T cells
Granulocytes
Identity
Ly6g
Itgam
Cd14
Cd3d
Cd3g
Cd3e
Ccr2
Fcgr3
Csf1r
Adgre1
Mrc1
Cd68
Afp
Krt18
Igfbp4
Nkg7
Prf1
Itga2
Col1a1
Loxl1
Lum
Fscn1
Flt3
Sirpa
Bst2
Cd19
Ms4a1
Cd79a
Pecam1
Cdh5
Erg
Features
Percent Expressed
0
25
50
75
Average Expression
2
1
0
-1
B
Cd14
S100a8
Ptgs2
Cd274
UMAP_1
UMAP_2
C
Elane
UMAP_1
UMAP_2

Figure S4

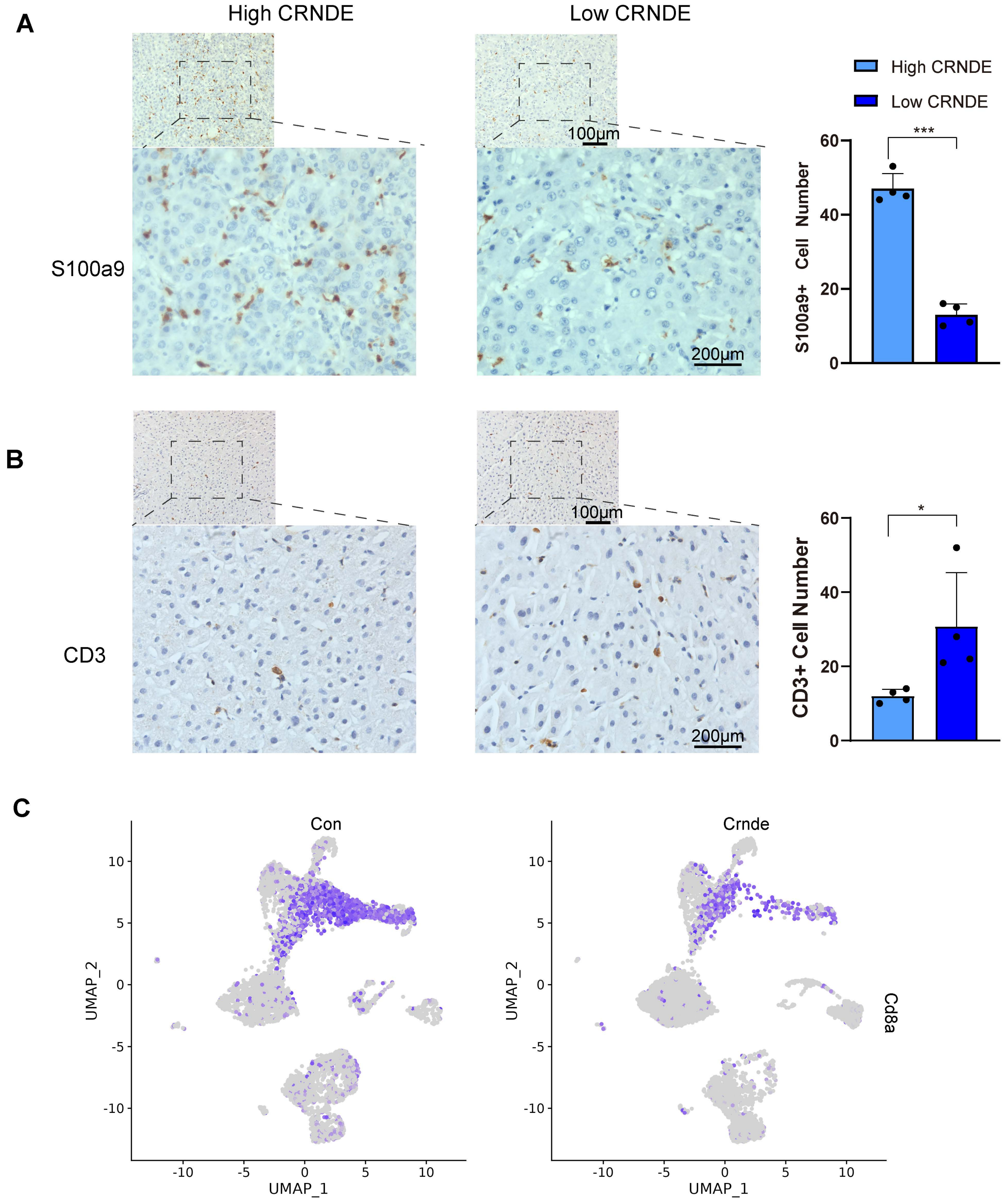
A
High CRNDE
Low CRNDE
S100a9
100μm
200μm
High CRNDE
Low CRNDE
S100a9+ Cell Number
***
0
20
40
60
B
CD3
100μm
200μm
CD3+ Cell Number
*
0
20
40
60
C
Con
Crnde
UMAP_1
UMAP_2
Cd8a
-10
-5
0
5
10

Figure S5

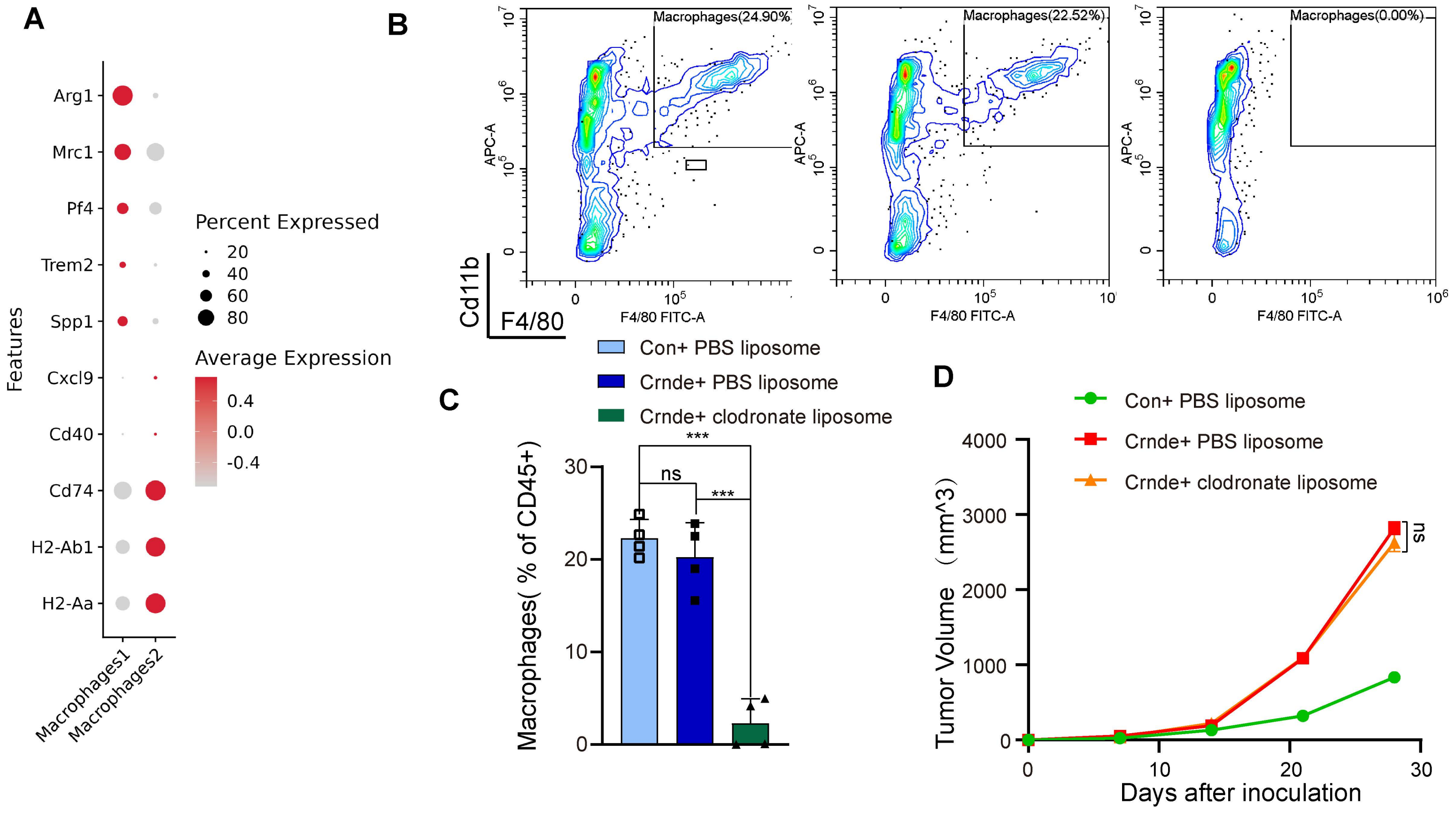
A
Arg1
Mrc1
Pf4
Trem2
Spp1
Cxcl9
Cd40
Cd74
H2-Ab1
H2-Aa
Features
Macrophages1
Macrophages2
Percent Expressed
20
40
60
80
Average Expression
0.4
0.0
-0.4
B
Con+ PBS liposome
Crnde+ PBS liposome
Crnde+ clodronate lipsome
Macrophages(24.90%
Macrophages(22.52%)
Macrophages(0.00%)
APC-A
F4/80 FITC-A
Cd11b
F4/80
C
Con+ PBS liposome
Crnde+ PBS liposome
Crnde+ clodronate liposome
Macrophages(% of CD45+)
ns
***
D
Con+ PBS liposome
Crnde+ PBS liposome
Crnde+ clodronate liposome
Tumor Volume （mm^3）
Days after inoculation
ns

Figure S6

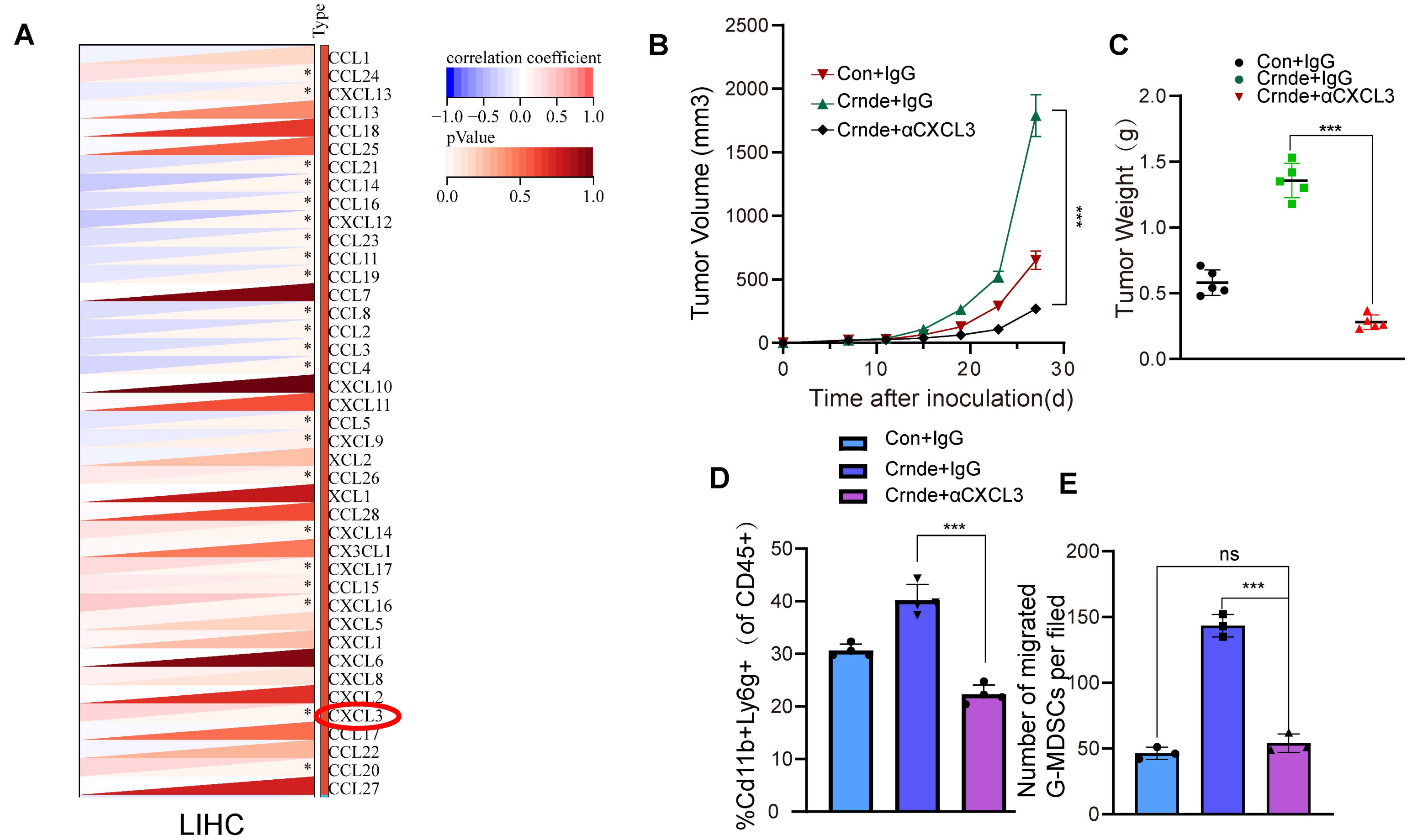

A
Type
CCL1
CCL24
CXCL13
CCL13
CCL18
CCL25
CCL21
CCL14
CCL16
CXCL12
CCL23
CCL11
CCL19
CCL7
CCL8
CCL2
CCL3
CCL4
CXCL10
CXCL11
CCL5
CXCL9
XCL2
CCL26
XCL1
CCL28
CXCL14
CX3CL1
CXCL17
CCL15
CXCL16
CXCL5
CXCL1
CXCL6
CXCL8
CXCL2
CXCL3
CCL17
CCL22
CCL20
CCL27
LIHC
correlation coefficient
−1.0 −0.5 0.0 0.5 1.0
pValue
0.0 0.5 1.0
B
Con+IgG
Crnde+IgG
Crnde+αCXCL3
Tumor Volume (mm3)
Time after inoculation(d)
***
C
Con+IgG
Crnde+IgG
Crnde+αCXCL3
Tumor Weight（g）
***
D
%Cd11b+Ly6g+（of CD45+）
***
E
Number of migrated G-MDSCs per filed
ns
***

Figure S7

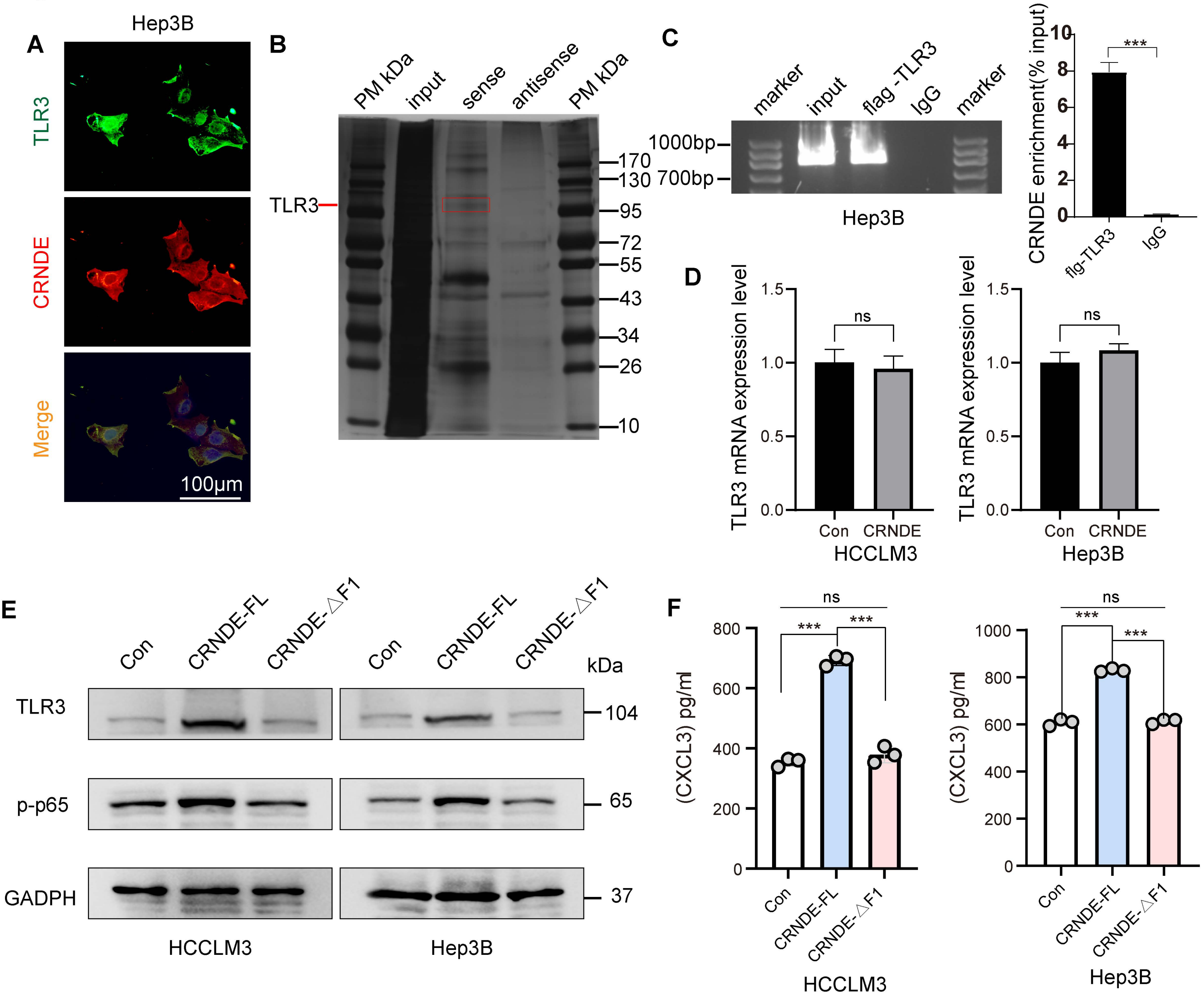

A
Hep3B
TLR3
CRNDE
Merge
100μm
B
PM kDa
input
sense
antisense
PM kDa
TLR3
170
130
95
72
55
43
34
26
10
C
marker
input
flag -TLR3
IgG
marker
1000bp
700bp
Hep3B
CRNDE enrichment(% input)
***
flg-TLR3
IgG
D
TLR3 mRNA expression level
ns
Con
CRNDE
HCCLM3
Hep3B
E
Con
CRNDE-FL
CRNDE-△F1
kDa
TLR3
p-p65
GADPH
104
65
37
HCCLM3
Hep3B
F
(CXCL3) pg/ml
ns
***
Con
CRNDE-FL
CRNDE-△F1
HCCLM3
Hep3B

Figure S8

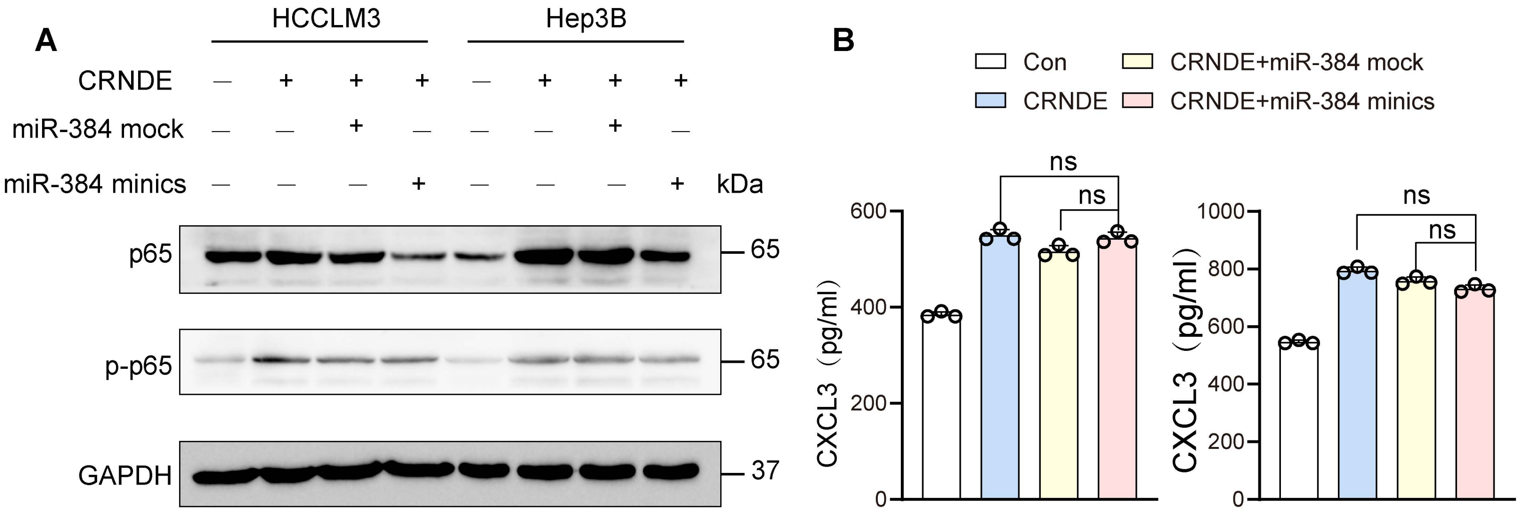
A
HCCLM3
Hep3B
CRNDE
miR-384 mock
miR-384 minics
kDa
p65
p-p65
GAPDH
65
65
37
B
Con
CRNDE
CRNDE+miR-384 mock
CRNDE+miR-384 minics
CXCL3（pg/ml）
ns
ns
600
400
200
0
1000
800
600
400
200
0

Supplementary Table1. Sequences of primers used in all experiments

Target	Sequence
GAPDH（H）	Forward: 5'-GTCTCCTCTGACTTCAACAGCG-3'
	Reverse: 5'-ACCACCCTGTTGCTGTAGCCAA-3'
Gapdh（M）	Forward: 5'-AGGTCGGTGTGAACGGATTTG-3'
	Reverse: 5'-GGGGTCGTTGATGGCAACA-3'
CRNDE（H）	Forward: 5'-AAATCAAAGTGCTCGAGTGGTT-3'
	Reverse: 5'-TAACCTTCTTCTGCGTGACAAC-3'
Crnde（M）	Forward: 5'-GGATAAGTGATGGCAGCAAGC-3'
	Reverse: 5'-CAGATGAACTCCTGTCTACGCC-3'
CXCL1	Forward: 5'-CTTGCCTCAATCCTGCATCC-3'
	Reverse: 5'-CTCTGCAGCTGTGTCTCTCT-3'
CXCL3	Forward: 5'-TTCACCTCAAGAACATCCAAAGTG-3'
	Reverse: 5'-TTCTTCCCATTCTTGAGTGTGGC-3'
CXCL6	Forward: 5'-AGAGCTGCGTTGCACTTGTT-3'
	Reverse: 5'-GCAGTTTACCAATCGTTTTGGGG-3'
CCL2	Forward: 5'-AGAATCACCAGCAGCAAGTGTCC-3'
	Reverse: 5'-TCCTGAACCCACTTCTGCTTGG-3'
TLR3	Forward: 5'-TTGCCTTGTATCTACTTTTGGGG-3'
	Reverse: 5'-TCAACACTGTTATGTTTGTGGGT -3'
CRNDE probe1	AGTTCAGAAGTGAACTTAGCTGACAGCATC
CRNDE probe2	GTCCGTTCGGCCCACGAGGGGACACGACTA
CRNDE probe3	AATCCTCAGTTGTCACGCAGAAGAAGGGGCA
Antisense probe1	CTACGACAGTCGATTCAAGTGAAGACTTGA
Antisense probe2	ATCAGCACAGGGGAGCACCCGGCTTGCCTG
Antisense probe3	ACGGGAAGAAGACGCACTGTTGACTCCTAA
CRNDE FL	Forward: 5'-GtcaagctaatacgactcactatagggTCTAGTCGTGTCCCCTCGTG-3'

	Reverse: 5'-TTCCTATACCTTGGCTAAAC-3'
CRNDE F1	Forward: 5'-GtcaagctaatacgactcactatagggTCTAGTCGTGTCCCCTCGTG-3'
	Reverse: 5'-CGACACCCGGAGAGGCTCGA-3'
CRNDE F2	Forward: 5'-GtcaagctaatacgactcactatagggCAATAGCCAGTACAGTAGCT-3'
	Reverse: 5'-CAATAGCCAGTACAGTAGCT-3'
CRNDE F3	Forward: 5'-GtcaagctaatacgactcactatagggCCTGTGATGTGTTTCAATCT-3'
	Reverse: 5'-TTCCTATACCTTGGCTAAAC-3'

Supplementary Table2. Antibody information

Antibody	Sourse	Cat. #
NF-κB p65	Cell Signaling Technology	82425
Phospho-NF-κB p65	Cell Signaling Technology	30335
NF-κB2 p100/p52	Cell Signaling Technology	48825
CD3	Proteintech	17617-1-AP
S100a9	Proteintech	26992-1-AP
PE anti-mouse CD45	BioLegend	147712
APC anti-mouse CD3	BioLegend	100236
PE anti-mouse/human CD11b Antibody	BioLegend	101208
APC/Fire™ 750 anti-mouse CD3	BioLegend	100248
F4/80 Monoclonal Antibody (BM8), FITC, eBioscience™	Thermo Fisher Scientific	4329362
APC/Fire™ 750 anti-mouse CD45	BioLegend	147714

CoraLite®488 Anti-Mouse Ly-6G	Proteintech	CL488-65140
TLR3 Rabbit pAb	ABclonal	A11778
Alexa Fluor™ 488 Goat anti-Mouse IgG (H+L)	Thermo Fisher Scientific	A-11001
Goat-anti-Rabbit IgG（H+L）	Vector laboratory	BA-1000
Rabbit (DA1E) mAb IgG XP® Isotype Control	Cell Signaling Technology	8726S
DYKDDDDK Tag (D6W5B) Rabbit mAb	Cell Signaling Technology	14793S
